# Supplementary material for: Multi‐dataset identification of innovative feature genes and molecular mechanisms in keratoconus
Source: J Cell Mol Med. 2024 Sep 19;28(18):e70079. doi: 10.1111/jcmm.70079 (PMC11412914; doi:10.1111/jcmm.70079)
Supplement: Supplementary file 2 — Material S2. [file JCMM-28-e70079-s002.zip › 7.GSEA analysis/1.ARL11/KEGG_TASTE_TRANSDUCTION.html]

Details for gene set KEGG\_TASTE\_TRANSDUCTION[GSEA]

|  || Dataset | ARL11.group.cls#h\_versus\_l |
| Phenotype | group.cls#h\_versus\_l |
| Upregulated in class | h |
| GeneSet | KEGG\_TASTE\_TRANSDUCTION |
| Enrichment Score (ES) | 0.33382246 |
| Normalized Enrichment Score (NES) | 0.9652232 |
| Nominal p-value | 0.52079207 |
| FDR q-value | 1.0 |
| FWER p-Value | 1.0 |
Table: GSEA Results Summary

  

Fig 1: Enrichment plot: KEGG\_TASTE\_TRANSDUCTION      
 Profile of the Running ES Score & Positions of GeneSet Members on the Rank Ordered List

  

| SYMBOL | TITLE | RANK IN GENE LIST | RANK METRIC SCORE | RUNNING ES | CORE ENRICHMENT || 1 | PLCB2 | na | 134 | 0.619 | 0.0691 | Yes |
| 2 | TAS2R4 | na | 402 | 0.501 | 0.1204 | Yes |
| 3 | TAS1R1 | na | 1206 | 0.365 | 0.1403 | Yes |
| 4 | GRM4 | na | 1776 | 0.313 | 0.1607 | Yes |
| 5 | TAS2R45 | na | 1867 | 0.306 | 0.1942 | Yes |
| 6 | CACNA1A | na | 2621 | 0.259 | 0.2029 | Yes |
| 7 | GNAT3 | na | 2636 | 0.258 | 0.2329 | Yes |
| 8 | TAS2R31 | na | 2698 | 0.255 | 0.2612 | Yes |
| 9 | TAS2R20 | na | 3165 | 0.233 | 0.2753 | Yes |
| 10 | TAS2R46 | na | 3428 | 0.222 | 0.2939 | Yes |
| 11 | TAS2R10 | na | 4209 | 0.193 | 0.2941 | Yes |
| 12 | TAS2R13 | na | 5258 | 0.158 | 0.2826 | Yes |
| 13 | TAS2R14 | na | 5305 | 0.157 | 0.2997 | Yes |
| 14 | CACNA1B | na | 5425 | 0.154 | 0.3144 | Yes |
| 15 | TAS2R9 | na | 5624 | 0.148 | 0.3261 | Yes |
| 16 | TAS2R40 | na | 6608 | 0.121 | 0.3121 | Yes |
| 17 | TAS1R3 | na | 6689 | 0.119 | 0.3238 | Yes |
| 18 | TAS2R1 | na | 6815 | 0.116 | 0.3338 | Yes |
| 19 | TAS2R43 | na | 8629 | 0.081 | 0.2910 | No |
| 20 | SCNN1A | na | 9359 | 0.069 | 0.2782 | No |
| 21 | TAS2R19 | na | 9454 | 0.068 | 0.2835 | No |
| 22 | PRKX | na | 9610 | 0.066 | 0.2868 | No |
| 23 | GNG3 | na | 10218 | 0.057 | 0.2760 | No |
| 24 | ADCY4 | na | 10290 | 0.056 | 0.2806 | No |
| 25 | ADCY6 | na | 10456 | 0.054 | 0.2823 | No |
| 26 | TAS2R42 | na | 10555 | 0.053 | 0.2857 | No |
| 27 | TAS2R3 | na | 10991 | 0.048 | 0.2787 | No |
| 28 | TAS2R38 | na | 11084 | 0.047 | 0.2816 | No |
| 29 | TAS2R39 | na | 13216 | 0.025 | 0.2231 | No |
| 30 | TAS2R8 | na | 14429 | 0.016 | 0.1900 | No |
| 31 | TAS2R60 | na | 14665 | 0.014 | 0.1848 | No |
| 32 | GNAS | na | 16257 | 0.002 | 0.1392 | No |
| 33 | TRPM5 | na | 16590 | -0.000 | 0.1296 | No |
| 34 | GNB1 | na | 17391 | -0.006 | 0.1073 | No |
| 35 | ITPR3 | na | 18064 | -0.011 | 0.0892 | No |
| 36 | PRKACB | na | 18969 | -0.017 | 0.0651 | No |
| 37 | TAS2R5 | na | 22406 | -0.044 | -0.0288 | No |
| 38 | TAS1R2 | na | 22577 | -0.046 | -0.0283 | No |
| 39 | SCNN1B | na | 22657 | -0.047 | -0.0251 | No |
| 40 | TAS2R7 | na | 23205 | -0.052 | -0.0347 | No |
| 41 | PRKACA | na | 23665 | -0.058 | -0.0411 | No |
| 42 | GNG13 | na | 24317 | -0.067 | -0.0520 | No |
| 43 | PRKACG | na | 25752 | -0.087 | -0.0832 | No |
| 44 | ADCY8 | na | 25957 | -0.090 | -0.0785 | No |
| 45 | ASIC2 | na | 28204 | -0.136 | -0.1272 | No |
| 46 | TAS2R50 | na | 31907 | -0.257 | -0.2038 | No |
| 47 | KCNB1 | na | 31914 | -0.257 | -0.1737 | No |
| 48 | PDE1A | na | 33895 | -0.404 | -0.1832 | No |
| 49 | TAS2R41 | na | 33937 | -0.410 | -0.1361 | No |
| 50 | GNB3 | na | 33964 | -0.415 | -0.0879 | No |
| 51 | TAS2R16 | na | 34242 | -0.461 | -0.0416 | No |
| 52 | SCNN1G | na | 34293 | -0.472 | 0.0125 | No |
Table: GSEA details [plain text format]

  

Fig 2: KEGG\_TASTE\_TRANSDUCTION      
 Blue-Pink O' Gram in the Space of the Analyzed GeneSet

  

Fig 3: KEGG\_TASTE\_TRANSDUCTION: Random ES distribution      
 Gene set null distribution of ES for **KEGG\_TASTE\_TRANSDUCTION**
